# Low efficiency of leucocyte plugging-based drug delivery to cancer in mice

**DOI:** 10.1007/s13346-021-01028-y

**Published:** 2021-07-28

**Authors:** Baifeng Qian, Andreas Termer, Christof M. Sommer, Arianeb Mehrabi, Eduard Ryschich

**Affiliations:** 1 Department of General, Visceral and Transplantation Surgery, University Hospital Heidelberg, Im Neuenheimer Feld 365, 69120 Heidelberg, Germany; 2 Clinic for Diagnostic and Interventional Radiology and Department of Nuclear Medicine; Clinic of Radiology, University Hospital Heidelberg, Heidelberg, Germany

**Keywords:** Leucocyte plugging, Trojan horse, Liver tumour, Mouse model

## Abstract

Cells of the immune system were proposed for use as Trojan horse for tumour-specific drug delivery. The efficacy of such cell-based drug delivery depends on the site-specific cell homing. This present study was aimed to investigate the potential of leucocytes for intratumoural site-specific enrichment using a locoregional application route in experimental liver tumours. Human neutrophils were isolated from peripheral blood and directly labelled with calcein AM or loaded with doxorubicin. The neutrophil loading and release of doxorubicin and the migration and adhesion to ICAM-1 were analysed in vitro. Macrophages were isolated and activated in vitro. Leucocyte plugging and the distribution pattern in the liver microvasculature were studied ex vivo, and the efficacy of leucocyte plugging in tumour blood vessels was analysed in vivo after superselective intra-arterial injection in mouse liver tumour models. Neutrophils were characterised by the high dose-dependent uptake and rapid release of doxorubicin. Doxorubicin loading did not affect neutrophil migration function. Neutrophil plugging in liver microvasculature was very high (> 90%), both after ex vivo perfusion and after injection in vivo. However, neutrophils as well as activated macrophages plugged insufficiently in tumour blood vessels and passed through the tumour microvasculture with a very low sequestration rate in vivo. Neutrophils possess several properties to function as potentially effective drug carriers; however, the tumour site-specific drug delivery after selective locoregional injection was observed to be insufficient owing to low intratumoural microvascular plugging.

**Graphical abstract:**

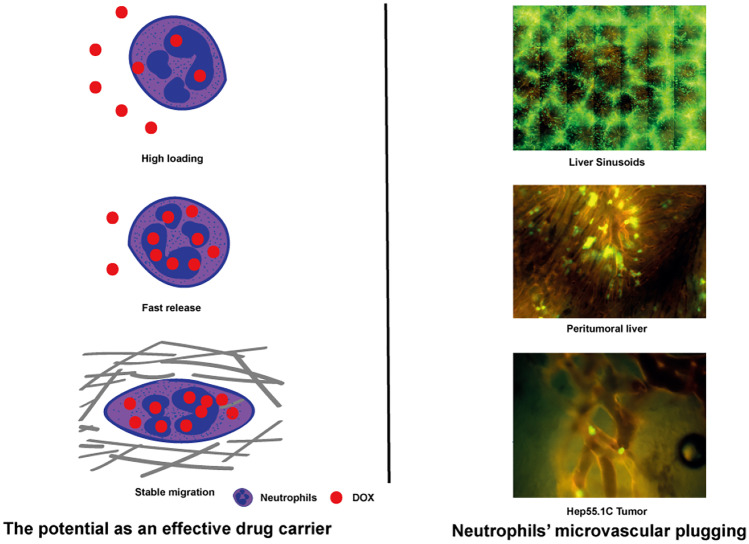

## Introduction

Cells belonging to the immune system, such as neutrophils [1], monocytes [2], dendritic cells [3, 4], macrophages [5] and lymphocytes [6] can serve as carriers to transport drugs to disease sites. These cells may potentially score over conventional routes of administration, because they prolong the half-life of the drug that they deliver and lower drug immunogenicity [7]. These cells can migrate across impermeable barriers (e.g. the blood-tumour barrier) for drug release. Some cells have been proposed for use as Trojan horses for drug delivery [8]. After systemic administration, immune cells can preferentially home to tumour sites; however, the number of cells homing to the tumour is too small for effective control of tumour growth [9, 10]. Therefore, novel strategies that improve cell homing are warranted to facilitate tumour-specific drug delivery by immunocytes.

Intravascular leucocyte sequestration [11] or intravascular plugging refers to the phenomenon of sequestration of circulating leucocytes in capillaries. Inflammatory signals can remodel the cell cytoskeleton, alter endothelial cell contractility and function and promote leucocyte sequestration in the microvasculature. Neutrophils constitute 50–70% of circulating leucocytes and represent the largest group of inflammatory cells in human blood [12]. Usually, they circulate freely in the blood but are rapidly activated by inflammatory signals [13]. They show chemotaxis and can migrate and extravasate to inflammatory tissue sites to defend the body against pathogenic microorganisms [14].

Studies have proved the efficacy of localised therapies, such as transarterial chemoembolisation and selective internal radiation therapy, which deliver high doses of cytotoxic drugs or radioactive beads to the tumour bed through intra-arterial application for advanced hepatic carcinoma, which could improve overall survival and limit systemic toxicity [15].

The present study investigated the role of neutrophil-based locoregional drug delivery as a potential Trojan horse for the treatment of liver tumours. Doxorubicin (DOX) was used as a chemotherapeutic agent, and intrahepatic and intratumoural neutrophil plugging was mapped and quantified after superselective locoregional administration.

## Materials and methods

### Isolation and cultivation of cells in vitro

For neutrophil isolation, buffy coats of healthy donors were diluted to 1:5 with phosphate-buffered saline (PBS), and cell pellets were obtained by density gradient centrifugation using 1.077 g/mL Biocoll (Biochrom, Berlin, Germany). The cell pellet was collected, and remnant erythrocytes were eliminated using an ammonium-chloride lysis buffer. Finally, neutrophils were isolated from the cell pellet using a Human CD15 Positive Selection Kit (Miltenyi Biotec, Bergisch Gladbach, Germany) based on positive immunomagnetic selection for cell separation, according to the manufacturer’s instructions.

For macrophage isolation and activation, buffy coats of healthy donor blood were diluted to 1:2 with PBS, and human peripheral blood mononuclear cells (PBMCs) were isolated following density gradient centrifugation using 1.077 g/mL Biocoll. PBMCs were treated with 100 ng/mL granulocyte–macrophage colony-stimulating factor (GM-CSF, GenScript, Piscataway, USA) for 48 h and for 5 days thereafter with 100 ng/mL GM-CSF, 50 ng/mL human recombinant γ-interferon (Affymetrix eBioscience, Wien, Austria), and 10 ng/mL lipopolysaccharides (Sigma-Aldrich, Taufkirchen, Germany) in phenol red RPMI-1640 medium supplemented with 10% foetal calf serum (FCS, CCPro, Oberdorla, Germany), 2 mM L-glutamine, 20 U/mL penicillin, 0.1 mg/mL streptomycin (CCPro), 1 mM sodium pyruvate, and 0.05 mM 2-mercaptoethanol (Thermo, Waltham, MA, USA). Following adherence, macrophages were harvested from the surface of the culture dish after 7 days.

Tumour cells (lines Panc02, Hep55.1C, BxPC-3, MIA PaCa-2, Hep3B, and HepG2) were grown in Iscove’s modified Dulbecco’s modified Eagle medium (DMEM) supplemented with 10% FCS, 2 mM L-glutamine, 20 U/mL penicillin, and 0.1 mg/mL streptomycin (all from CCPro) at 37 °C in a humidified atmosphere with 5% carbon dioxide (CO_2_).

### Cytotoxic effects of doxorubicin on human and murine tumour cells

Cell viability after treatment with DOX was evaluated using a resazurin assay [16]. Cells were plated in 96-well plates (Greiner Bio-One, Frickenhausen, Germany) (300 μL/well). The initial cell number was adjusted to form a dense but non-confluent cell monolayer 24 h after seeding. The Panc02 and Hep55.1C cells were seeded at a concentration of 5000 cells/200 μL; the BxPC-3, MIA PaCa-2, and HepG2 cells at a concentration of 10,000 cells/100 μL; and the Hep3B cells at 6000 cells/100 μL. After a 24-h incubation, the cell medium was replaced with a fresh medium containing different DOX concentrations (Sigma-Aldrich, Taufkirchen, Germany), maleimide-functionalised doxosomes (MLP-DOX, Encapsula NanoSciences, Brentwood, CA, USA), or Caelyx (Schering-Plough, Kenilworth, NJ, USA) (range 0–30 μM). After a 24-h incubation, the medium containing DOX was replaced with one containing 10% resazurin. After a 4-h incubation, we measured absorption at 570 nm or fluorescence at 544 nm excitation and 590 nm emission. The half-maximal inhibitory concentration of DOX and 90% of the maximal inhibitory concentration of DOX were calculated using the SPSS software, version 21 (IBM, Armonk, NY, USA).

### Leucocyte loading with doxorubicin

Using different DOX concentrations (2.34, 4.69, 9.38, 18.75, 37.5, 75, and 150 µM) and different time intervals (15, 30, and 60 min), we incubated 2 × 10^6^ leucocytes (neutrophils or macrophages) at 37 °C. After washing with PBS, fluorescence of the cell suspension was directly measured using a fluorimeter (FLUOstar OPTIMA, BMG Labtech, Ortenberg, Germany) at 485 nm excitation and 580 nm emission.

### Doxorubicin release from neutrophils in vitro

The time course of the fluorescence signal change was analysed to study the release of DOX from neutrophils. After loading with DOX, neutrophils were incubated in the medium at 37 °C under continuous rotation (MACS rotatory mixer, Miltenyi Biotec, Bergisch Gladbach, Germany). After incubation for 30 or 60 min, the medium was removed, and cells were washed using PBS. The mean integral density of the fluorescence was measured in at least 15 neutrophils using a fluorescence microscopy system equipped with a monochromatic light source (excitation 470 nm and emission 580 nm) and control software (Axio Observer Z1, Zeiss, Jena, Germany). Fluorescence signal values were analysed using image-based fluorimetry (ImageJ, Bethesda, MD, USA), corrected for background and expressed as the mean fluorescence intensity. DOX release was calculated as loss of the baseline fluorescence and was expressed as a percentage.

### Migration of neutrophils in the three-dimensional collagen matrix

Leucocyte migration in the three-dimensional (3D) collagen matrix was investigated as previously described [17]. The collagen matrix was prepared using 1700 μL of 0.2% collagen R (Serva, Heidelberg, Germany), 266 μL medium (RPMI 1.04 g [Merck, Darmstadt, Germany]) + 240 mg HEPES (PAA Laboratories, Cölbe, Germany) + 200 mg bovine serum albumin (BSA, New England Biolabs, Frankfurt a.m., Germany) in 10 mL PBS, 110 μL sodium hydroxide (0.34 M), and 200 μL of cell suspension (concentration of 2–20 × 10^6^/mL) [17]. The fresh collagen solution was gently mixed in a Petri dish (Greiner Bio-One) by circular rotation for 30 s and was allowed to polymerise for 30 min at 37 °C. Cell migration was recorded in a time-lapse manner using a 60-s interval for 20–30 min directly after polymerisation using a microscopy system (Axio Observer Z1). Migration was analysed using the manual tracking function in ImageJ (National Institutes of Health). The movement velocities of 20 active cells in each experiment were analysed. Ketoprofen (4 mM [CT Arzneimittel, Berlin, Germany]) was added to the collagen matrix to suppress leucocyte migration [18]. Additionally, phototoxicity-induced cell immobilisation [19, 20] was achieved using irradiation with a curing light source (430–480 nm, 1.5 W/cm^2^, M&W Dental, Büdingen, Germany) of calcein-acetoxymethyl (AM)–labelled neutrophils (5 mm distance to the light source, irradiation time 12 s).

### Human neutrophil adhesion to intercellular adhesion molecule-1

Microfluidic chambers (μ-Slide VI 0.4, Ibidi, Martinsried, Germany) were coated with human intercellular adhesion molecule-1 (ICAM-1) (13.3 μg/mL, PeproTech, Rocky Hill, USA), murine ICAM-1 (13.3 μg/mL, R&D Systems, Minneapolis, USA) or were covered with only PBS (control group) for 2 h and subsequently blocked using 10% BSA solution for 1 h at room temperature. To study the binding of immune cells to the immobilised ICAM-1, 100 μL of the cell suspension (3 × 10^6^ cells/mL) per flow chamber were incubated for 5 min at room temperature. Non-bound cells were removed using flow perfusion with PBS at a perfusion rate of 5.7 mL/min (approximately 7.25 dyn/cm^2^) and a perfusion volume of 1 mL. Finally, the number of adhered human neutrophils was counted using microscopy (Axio Observer Z1) and expressed per square millimetre.

### Tumour induction and superselective injection

All animal experiments were performed in accordance with international rules and were approved by the local committee of animal care (Regierungspräsidium Karlsruhe), and we used C57BL/6 mice (males, 8–12 weeks old, Charles River, Sulzfeld, Germany) for this study. The animals were housed in standard cages, were exposed to a 12-h light–dark cycle and received standard animal feed. Intrahepatic inoculation of 1.5–2 × 10^6^ tumour cells into the left lateral lobe was performed to induce tumours for the in vivo experiment [21], that led to the development of solid well-vascularised tumours at the inoculation site. Mouse pancreatic cancer (Panc02) or hepatocellular cancer cell lines (Hep55.1C) were used as previously described [22, 23]. Superselective intra-arterial injections were performed 12 − 14 days after inoculation, as previously described [22, 23].

### Human neutrophil sequestration in the liver microvasculature

Isolated mouse liver perfusion was performed as previously described to analyse the efficacy of human neutrophil sequestration in the liver microvasculature [23, 24]. Briefly, human neutrophils were treated with 4 μg/mL calcein-AM (Santa Cruz Biotechnology, Dallas, TX, USA) for 15 min, washed, and filtered through 35-μm cell strainers (Corning, Reynosa, Tamaulipas, Mexico). After euthanising the mice and opening the abdominal cavity, a thin catheter (∅ 0.61 mm, Reichelt Chemietechnik, Heidelberg, Germany) was inserted into the portal vein and connected to a syringe infusion pump (WPI, Sarasota, FL, USA). Blood from the liver was removed using 1 mL/min perfusion with PBS. We infused 2 mL of PBS containing 800 ng of R-phycoerythrin (RPE)-conjugated ME-9F1 mAb (BioLegend, San Diego, CA, USA) at a flow rate of 1 mL/min for microvascular imaging. The liver was removed and placed into a small Teflon chamber (Neolab, Heidelberg, Germany). Using the infusion pump, 1.5 mL of the cell suspension (3 × 10^6^ cells/mL) was injected at a defined perfusion rate (0.5, 1, and 3 mL/min). Finally, images of the visceral and diaphragmatic aspects and a cross-sectional view were obtained using a fluorescence microscopy system (Axio Observer.Z1) (excitation 488 nm for calcein-AM and 555 nm for RPE). Intermittent liver perfusion [24] was performed using intermittent “perfusion-effluent collection” cycles with 1 × 10^6^ cells/cycle at a 1-mL/min flow rate. Neutrophils in the effluent were counted, and the efficacy of neutrophil sequestration in the liver microvasculature was calculated as ratio/percentage of cells which did not pass and retained in the liver. Additionally, leucocyte plugging was visualised using a fluorescence microscopy system as described above.

### Leucocyte sequestration in a tumour-bearing mouse model

Human calcein-AM-labelled neutrophils were prepared for perfusion as described earlier. Selective injection of the labelled cells into the hepatic artery was performed as previously described [25]. Briefly, tumour-bearing mice were anaesthetised using intraperitoneal injections of 40 mg/kg ketamine (Pfizer, Berlin, Germany) and 10 mg/kg xylazine (CP-Pharma, Burgdorf, Germany). The abdominal cavity was opened, and adhesions between the duodenum and liver were carefully released. Selective injection into the hepatic artery was performed using a 34-G needle with a 25-µL microsyringe (Hamilton, Bonaduz, Switzerland) using the next distal branch following the branching site of the hepatic artery.

After all branches of the hepatic artery were ligated except an artery feeding the tumour-bearing liver area, selective hepatic arterial injection of 25 μL of 4.5–5.5 × 10^6^ calcein-AM-labelled neutrophils or macrophages was performed into the tumour-feeding artery. Under sterile conditions, the animal with the exposed tumour was placed in the prone position on the cover slip of the temperature-controlled (37 °C) chamber. Using a fluorescence microscope (Axio Observer Z1), time-lapse microscopy of the same tumour/liver area was performed at the aforementioned excitation with a time interval of 20 s [22]. The mice were sacrificed 5–10 min after injection, and organs were removed for imaging. The whole-mount tissue (tumour, peritumoural liver, lung, spleen) was imaged using a fluorescence microscopy system (Axio Observer.Z1) as described above. The number of sequestrated leucocytes was counted using ImageJ. The highest sequestration (peritumoral liver) was used as reference for calculation of percentage in other tissues.

## Results

### Cytotoxic effect of different doxorubicin formulations on human and murine tumour cells

**Fig. 1:**
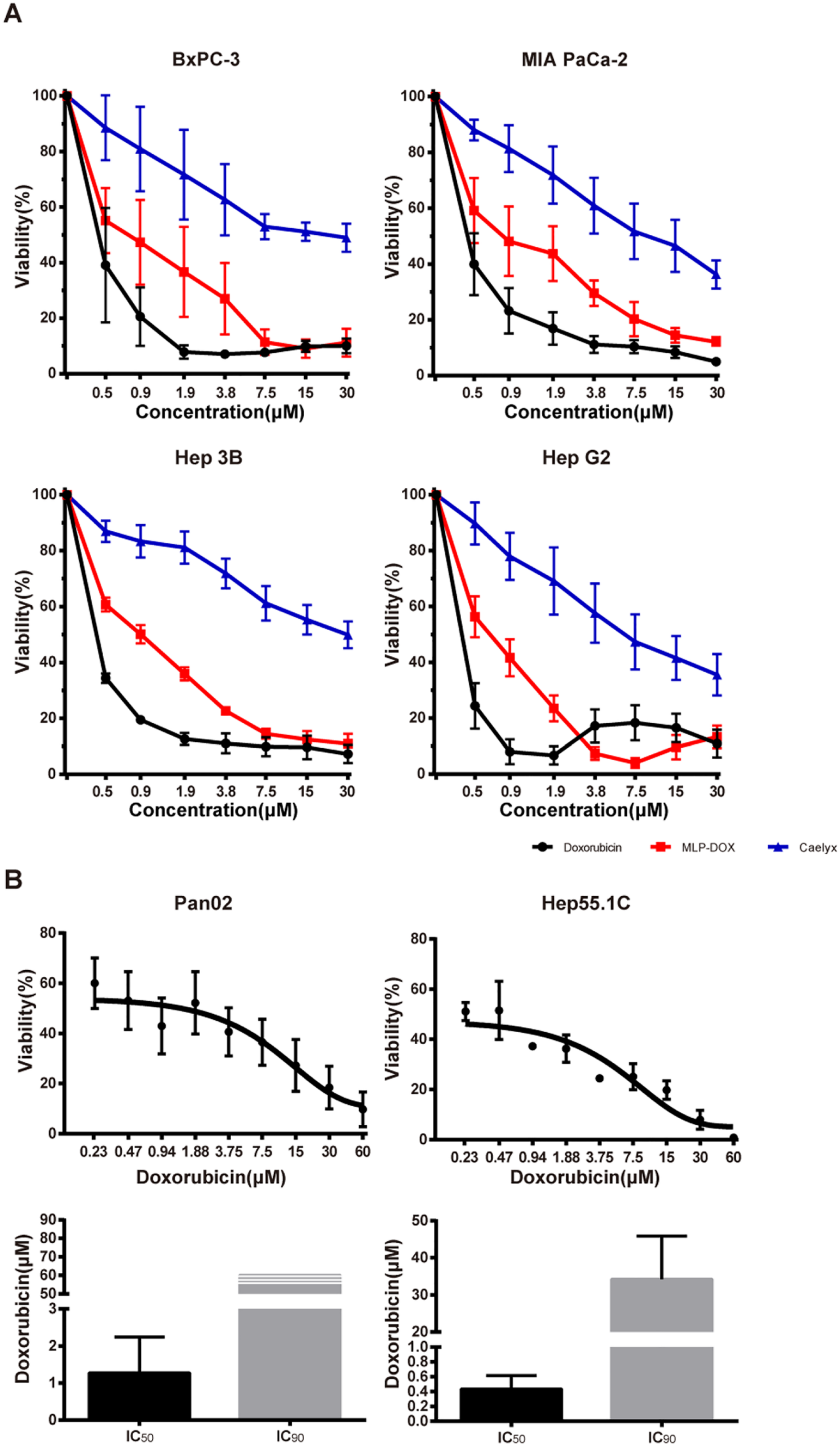
**A** Dose–effect relationship in the resazurin viability assay for human tumour cell lines BxPC-3, MIA PaCa-2, Hep3B and HepG2. In all tumour cell lines, a comparably high effectiveness of doxorubicin and MLP-DOX compared to Caelyx is shown. **B** Cytotoxic effect of doxorubicin in Panc02 and Hep55.1C. The IC50 and IC90 of doxorubicin in Panc02 and Hep55.1C are shown at 24 h

Three different DOX formulations (one pure and two encapsulated [liposomal] DOX types) were analysed to identify the best DOX preparation for neutrophil loading. Pure DOX showed the strongest dose- and time-dependent cytotoxic effects in all human (Fig. 1A) and murine (Fig. 1B) cell lines. The cytotoxic effect of surface-activated DOX (MLP-DOX) was higher than that of pegylated liposomal DOX (Caelyx). Non-capsulated DOX was selected for subsequent analyses owing to its high efficacy.

### High uptake and high spontaneous release of doxorubicin by neutrophils

**Fig. 2:**
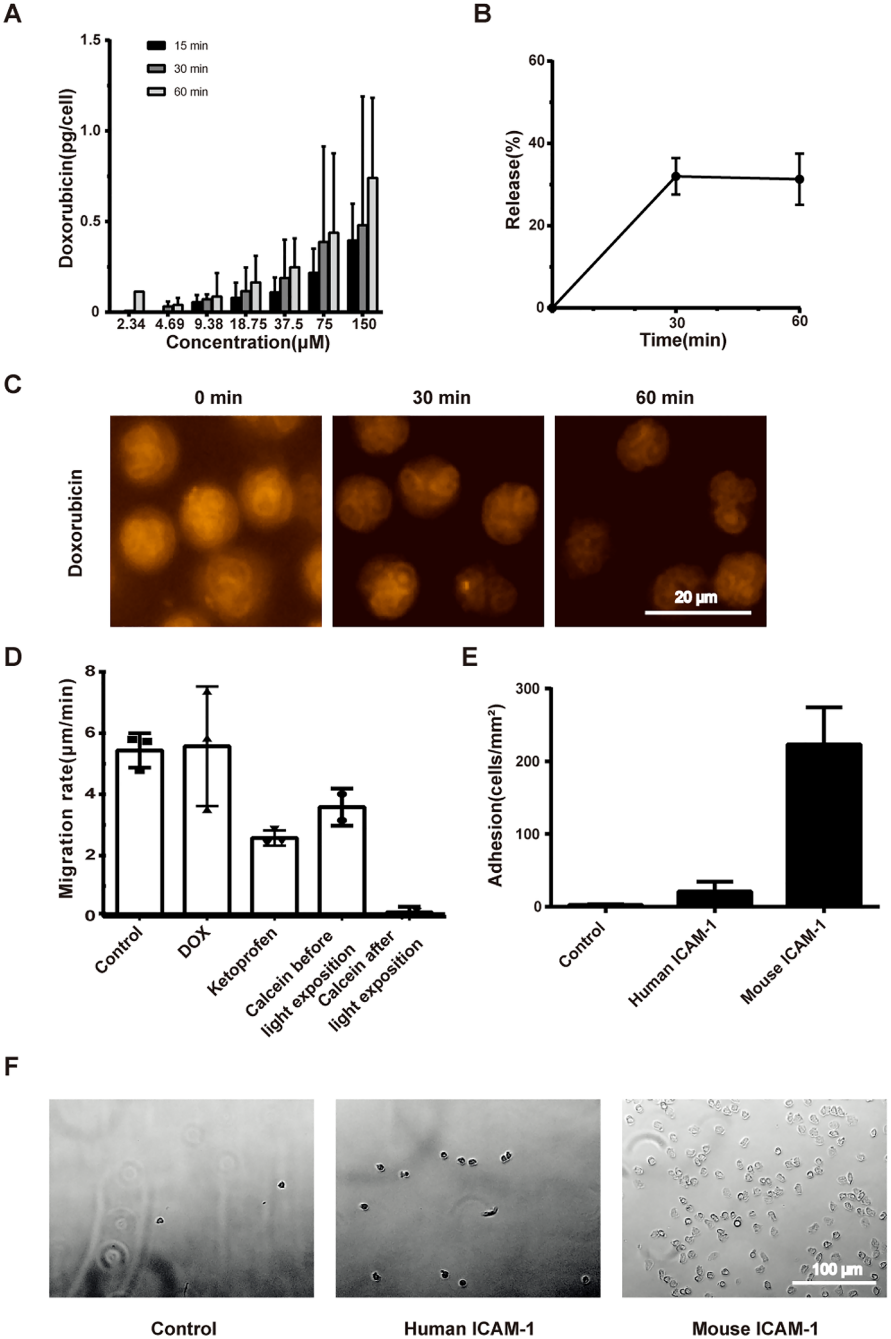
**A** The doses of neutrophil loading with doxorubicin at different conditions. **B** Spontaneous doxorubicin release from neutrophils in vitro. **C** The representative images of doxorubicin release from neutrophils. **D** The migration velocity of neutrophils at different conditions. **E** The efficacy of neutrophil adhesion to solid-phase mouse and human ICAM-1, compared with non-coated. **F** The representative images of neutrophil adhesion to coated solid-phase ICAM-1

A high uptake capacity and rapid release of the cargo substance are important prerequisites for the effective function of cell plugging-based drug delivery. We determined the uptake capacity of neutrophils by incubating neutrophils with different DOX concentrations at different incubation times and observed dose- and time-dependent loading of DOX in neutrophils; the higher the incubation concentration and the longer the incubation time, the higher the DOX uptake by neutrophils. The intracellular concentration of DOX in neutrophils was 0.74 ± 0.44 pg/cell (Fig. 2A) with an incubation time of 60 min and a constant concentration (150 μM). Analysis of DOX release showed that intracellular fluorescence (DOX content) significantly reduced within the first 30 min (DOX release of 32 ± 4%) and did not show any change after 30 min (Fig. 2B, C).

### Neutrophil migration in a three-dimensional collagen matrix

Neutrophils are actively mobile cells that can extravasate and migrate into tissues. The potential change in neutrophil migration after DOX loading was measured using a 3D collagen matrix to predict cell behaviour after treatment. Additionally, neutrophil immobilisation would theoretically prolong intravascular persistence and microvascular obstruction. To study this mechanism, we used the following approaches: (a) treatment with ketoprofen [18] and (b) immobilisation using calcein-AM phototoxicity. No significant differences were observed in the migration rates of neutrophils between the control and DOX groups (5.44 ± 0.57 μm/min and 5.57 ± 1.95 μm/min, respectively, Fig. 2C). Ketoprofen and calcein alone induced a reduction in neutrophil migration (2.50 ± 0.29 μm/min and 3.58 ± 0.61 μm/min, respectively) (Fig. 2D), whereas calcein phototoxicity led to near-complete cell immobilisation (0.14 ± 0.18 μm/min) (Fig. 2D).

### Isolated neutrophils show high adhesive potential to solid-phase intercellular adhesion molecule-1

ICAM-1 is one of the most important adhesion molecules that control neutrophil adhesion to the endothelium [26]. To study the adhesive potential of neutrophils after isolation, we analysed neutrophil adhesion to human and mouse ICAM-1 under flow. We observed that both human and murine solid-phase ICAM-1 significantly promoted adhesion of human neutrophils (Fig. 2E, F). The adhesion to mouse ICAM-1 was even higher than that of human ICAM-1 (Fig. 2E, F).

### Neutrophil plugging in liver microvasculature ex vivo

**Fig. 3:**
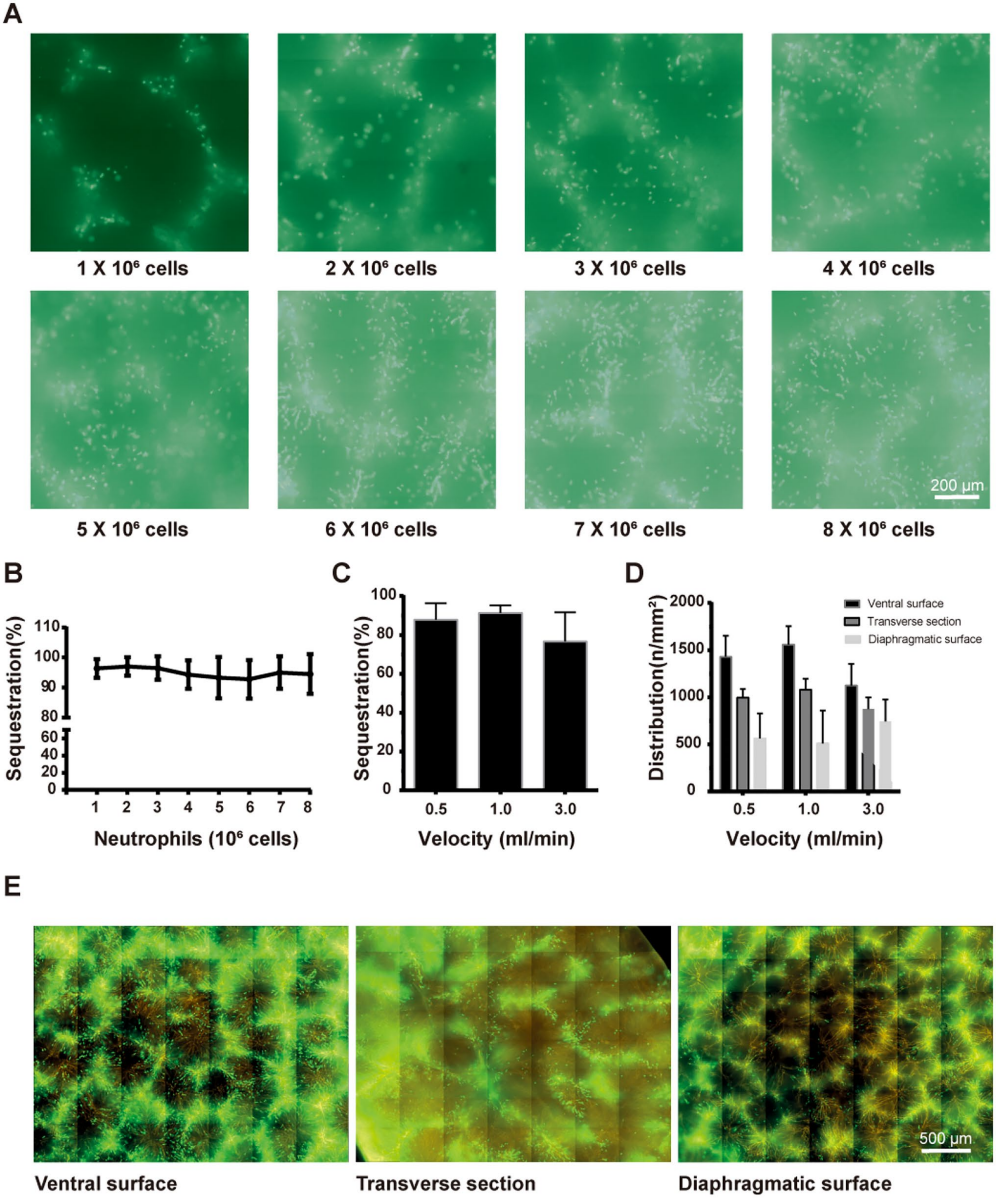
**A** Representative images show neutrophil sequestration after subsequent cycles of perfusion ex vivo. **B** The neutrophil sequestration efficiency after subsequent cycles of perfusion ex vivo. **C** The neutrophil sequestration efficiency at different perfusion velocities ex vivo expressed as percentage of intrahepatic retained cells. **D** The neutrophil distribution in the liver at different perfusion velocities ex vivo. **E** Representative images show neutrophil distribution in the liver ex vivo. The vessels were labelled with RPE-conjugated ME-9F1 mAb (orange), and monocytes were labelled with calcein AM (green)

Intrahepatic microvascular plugging with labelled neutrophils was investigated using an isolated liver perfusion model [23, 24]. We observed that only few neutrophils passed freely through the liver microvasculature. Most neutrophils plugged and obstructed the microvasculature (Fig. 3A, B). The increase in perfusion flow showed no or a minimal effect on hepatic microvascular neutrophil plugging (Fig. 3C). An increase in the amount of injected neutrophils was clearly accompanied by an increase in the leucocyte mass in the liver microvasculature (Fig. 3A, B). The fraction of plugged neutrophils was stable (approximately 95%) up to 8 × 10^6^ injected cells (Fig. 3B). Interestingly, the fraction of plugged leucocytes decreased to 82% after injection of 15 × 10^6^ cells. The amount of plugged neutrophils at the visceral site of the liver was significantly higher than that at the diaphragmatic site (Fig. 3D, E).

### Leucocyte plugging in liver microvasculature in vivo

**Fig. 4:**
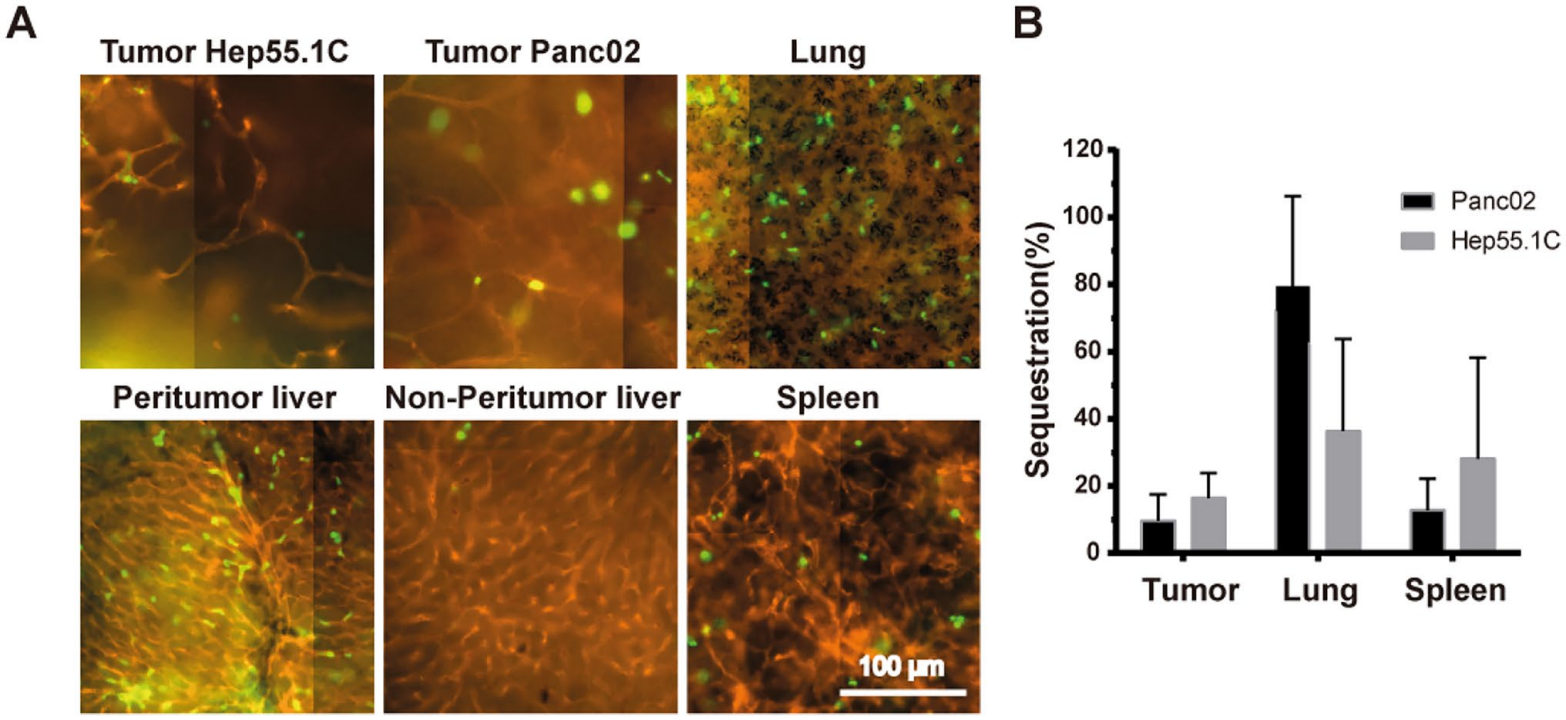
**A** Representative imaging of neutrophil plugging in Panc02 and Hep55.1C tumour-bearing mouse. **B** Comparison of the number of sequestered neutrophils in the tumour, lung and spleen expressed as percentage of sequestrated neutrophils in relation to the peritumoral liver tissue

Selective hepatic artery injection was performed to evaluate in vivo neutrophil plugging in tumour-bearing mice. The liver vessels were stained with a PE-conjugated anti-CD146 (ME-9F1) antibody, and the injected neutrophils were loaded with calcein-AM before perfusion and were clearly recognisable using fluorescence microscopy. Peritumoural liver tissue showed neutrophil sequestration that showed largely inhomogeneous cell distribution, mainly in the liver sinusoids. In the non-peritumoural liver tissue, which was excluded from the perfusion by vessel ligation, we observed only isolated neutrophils. Furthermore, neutrophils were rarely detected in tumour-associated microvessels in both Panc02 and Hep55.1C tumours (Fig. 4A).

Interestingly, we observed high intrapulmonary microvascular neutrophil plugging (Fig. 4A, B), as well as numerous plugged neutrophils in the splenic microvessels (Fig. 4A, B).

**Fig. 5:**
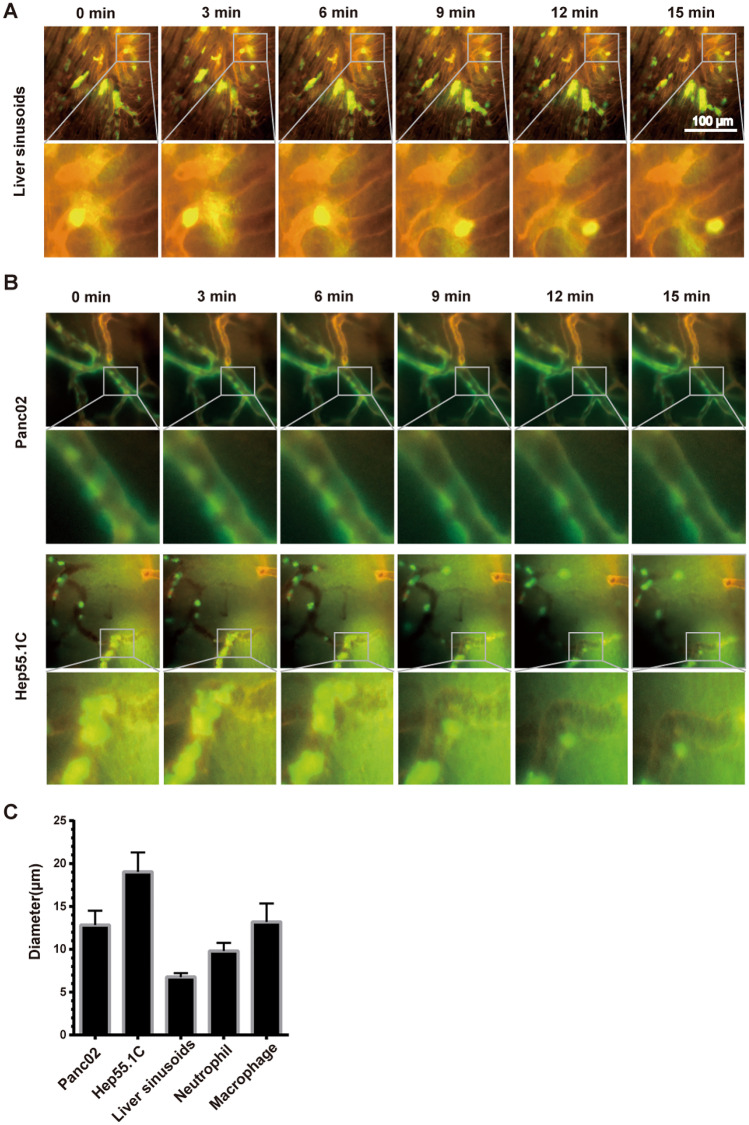
**A**, **B** Representative examples of time sequences of intravascular neutrophil migration crawling of neutrophil in liver sinusoids (**A**) and in tumour blood vessels (**B**). The vessels were labelled with RPE-conjugated ME-9F1 mAb (orange), and neutrophils were labelled with calcein AM (green). **C** The diameter of vessels of Panc02 tumour, Hep55.1C tumour, liver sinusoids and the diameter of neutrophils and macrophages

In vivo time-lapse microscopy was performed directly after cell injection to investigate the real-time process of neutrophil adhesion and plugging in tumour microvessels, as well as in presinusoidal vessels. Using this technique, numerous plugged leucocytes were detected in the presinusoidal blood vessels that completely occluded the vessel lumen (Fig. 5A). They also showed active intravascular crawling [17] and persisted over the entire duration of observation (15 min) (Fig. 5A). Few adherent neutrophils were also identified in tumour-associated blood vessels in both Panc02 and Hep55.1C tumours. The adhered neutrophils did not obstruct the lumen of a single blood vessel because of the higher blood vessel diameter (Fig. 5C). These cells underwent detachment from the endothelium after several minutes of adhesion and re-entered the circulation (Fig. 5B). Only individual neutrophils remained adherent at the end of the observation period, and no intravascular crawling [17] or extravasation of cells was detected (Fig. 5B).

**Fig. 6:**
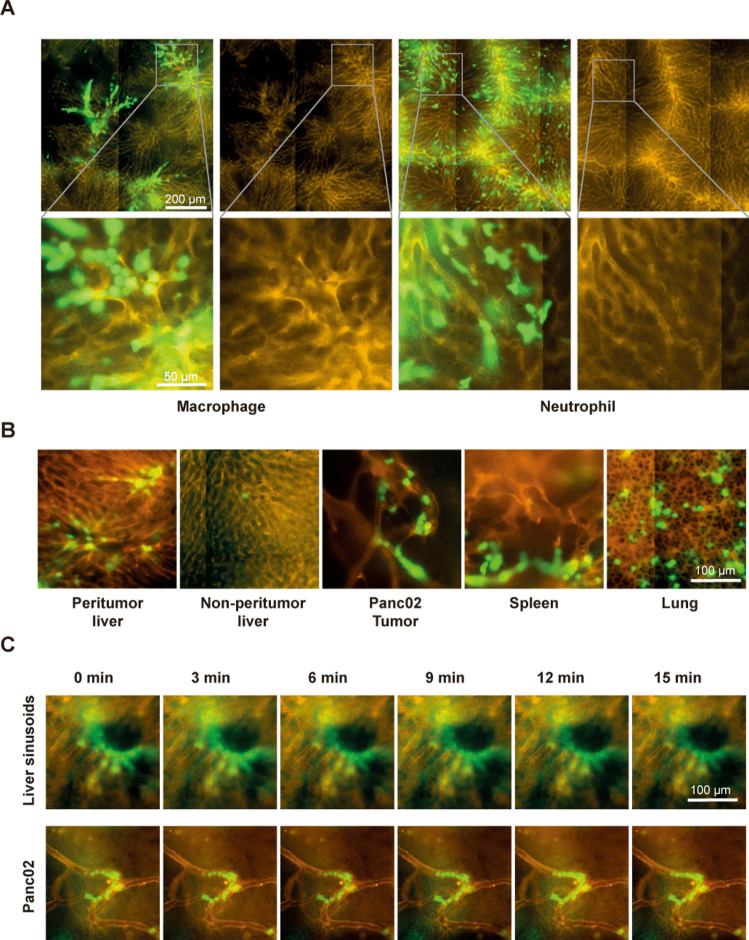
**A** Microvascular plugging patterns of macrophages and neutrophils in an ex vivo model. **B** Overview of macrophage sequestration in Panc02 tumour-bearing mice. **C** Fluorescence microscopic time-lapse sequence of the intravascular macrophage crawling in liver sinusoids and Panc02 tumour

We performed ex vivo and in vivo experiments using labelled macrophages to improve leucocyte plugging in tumour-associated blood vessels and to compensate for the difference between leucocyte size and vessel diameter. In isolated liver perfusion experiments, macrophages were plugged in the larger presinusoidal vessels rather than in the liver sinusoids (Fig. 6A). In vivo selective intra-arterial injection did not lead to a significant increase in leucocyte plugging in tumour blood vessels, although we observed a higher number of macrophages than neutrophils (Figs. 4A and 6B). Both the spleen and lungs showed marked macrophage sequestration (Fig. 6B). Time-lapse microscopy revealed no active locomotion of macrophages both in the liver sinusoids and in the tumour microvessels (Fig. 6C).

## Discussion

In the present study, DOX showed high cytotoxic effects in both human tumour cell lines (BxPC-3, MIA PaCa-2, Hep3B, and HepG2) and murine tumour cell lines (Panc02 and Hep55.1C). These effects were dose- and time-dependent, and our results concur with those of previous studies reported by Yagublu et al. [27] and Bour et al. [28].

In this study, neutrophils served as a Trojan horse to load and release DOX and showed sequestration in the liver microvasculature, and this method may be viewed as a promising approach for effective drug delivery. Neutrophil loading with DOX depends on differences in gradient concentrations between intra- and extracellular DOX. In our study, each neutrophil was loaded with 0.74 ± 0.44 pg of DOX with an incubation time of 60 min and a constant concentration (150 μM). Each cell spontaneously released approximately 32% DOX in the first 30 min in vitro; however, after 30 min, nearly no DOX was released from the neutrophils, which indicated the quantity of bound DOX. Based on the aforementioned effective loading and dynamics of release, neutrophils represent an effective carrier system for DOX; however, they must be immediately transported to the site of action owing to the short time required for effective DOX release.

The present study proved that freshly isolated neutrophils actively migrate both in a 3D collagen matrix in vitro and in liver sinusoids in vivo, and DOX loading does not affect neutrophil migration. If necessary, effective neutrophil immobilisation can be achieved using the phototoxic effect of calcein-AM or ketoprofen. Calcein-based phototoxicity is known to induce intracellular injury through the release of free radicals [19, 20], and ketoprofen induces reversible immobilisation [18]. Excessive light energy can be applied to immobilise neutrophils in vitro. As proved by the present study, a low degree of light energy is required for microscopy of calcein-labelled cells in vivo; we observed that it did not lead to cell immobilisation, and stable neutrophil migration in liver microvessels could be documented.

Isolated human neutrophils showed high adhesion to solid-phase murine and human ICAM-1 in vitro. This may represent a potentially important mechanism and a prerequisite for effective intravascular plugging after selective locoregional administration of human cells in a mouse model, which was subsequently confirmed by near-complete neutrophil plugging in hepatic blood vessels in an isolated liver perfusion model. This model also showed that the liver has a high but not unlimited capacity for intravascular leucocyte accumulation. Intrahepatic leucocyte plugging is practically independent of the perfusion rate. The difference in neutrophil distribution between the ventral and diaphragmatic sites may indicate the segmental liver structure and segment-specific macrovascular anatomy.

We observed significantly high neutrophil plugging in the peritumoural liver in vivo, although tumour blood microvessels do not support intravascular leucocyte accumulation. We propose that this finding can primarily be attributed to the increased diameter of tumour microvessels, which exclude additional mechanical factors that usually support microvascular neutrophil plugging. The difference between tumour microvessel diameter and injected cells is high. As shown in the present study and confirmed by previous investigations [24], the use of even larger leucocytes (macrophages) cannot significantly improve intratumoural microvascular plugging.

Usually, in healthy individuals, leucocytes circulate in the blood stream and do not plug blood vessels. However, under inflammatory conditions, leucocytes are activated, adhere to the endothelium of small venules, and migrate to the site of inflammation. Neutrophils can also plug capillaries to form dense intracapillary accumulations, which are previously described in the pancreatic [29] and hepatic [30] microvasculature. In the current study, no special measures were used for neutrophil activation. Neutrophils are sensitive to conditions during isolation and undergo activation, as proved by in vitro (adhesion assay), ex vivo (isolated liver perfusion), and in vivo (intravascular plugging) experiments.

In summary, leucocytes possess several properties to function as potentially effective drug carriers; however, in this study, tumour site-specific drug delivery after selective locoregional injection was observed to be insufficient owing to low intratumoural microvascular plugging.

## Data Availability

The datasets generated and analysed during the current study are available from the corresponding author on reasonable request.

## References

[1] Chu DF, Dong XY, Zhao Q, Gu JK, Wang ZJ (2017). Photosensitization priming of tumor microenvironments improves delivery of nanotherapeutics via neutrophil infiltration. Adv Mater.

[2] Lee S (2014). Monocytes: a novel drug delivery system targeting atherosclerosis. J Drug Target.

[3] Banchereau J, Palucka AK (2005). Dendritic cells as therapeutic vaccines against cancer. Nat Rev Immunol.

[4] Nencioni A, Grunebach F, Schmidt SM, Muller MR, Boy D, Patrone F, Ballestrero A, Brossart P (2008). The use of dendritic cells in cancer immunotherapy. Crit Rev Oncol Hemat.

[5] Choi MR, Stanton-Maxey KJ, Stanley JK, Levin CS, Bardhan R, Akin D, Badve S, Sturgis J, Robinson JP, Bashir R, Halas NJ, Clare SE (2007). A cellular Trojan horse for delivery of therapeutic nanoparticles into tumors. Nano Lett.

[6] Stephan MT, Moon JJ, Um SH, Bershteyn A, Irvine DJ (2010). Therapeutic cell engineering with surface-conjugated synthetic nanoparticles. Nat Med.

[7] Pang L, Zhang C, Qin J, Han LM, Li RX, Hong C, He HN, Wang JX (2017). A novel strategy to achieve effective drug delivery: exploit cells as carrier combined with nanoparticles. Drug Deliv.

[8] Batrakova EV, Gendelman HE, Kabanov AV (2011). Cell-mediated drug delivery. Expert Opin Drug Deliv.

[9] Basel MT, Balivada S, Wang H, Shrestha TB, Seo GM, Pyle M, Abayaweera G, Dani R, Koper OB, Tamura M, Chikan V, Bossmann SH, Troyer DL (2012). Cell-delivered magnetic nanoparticles caused hyperthermia-mediated increased survival in a murine pancreatic cancer model. Int J Nanomedicine.

[10] Li S, Feng S, Ding L, Liu Y, Zhu Q, Qian Z, Gu Y (2016). Nanomedicine engulfed by macrophages for targeted tumor therapy. Int J Nanomedicine.

[11] Doerschuk CM, Mizgerd JP, Kubo H, Qin L, Kumasaka T (1999). Adhesion molecules and cellular biomechanical changes in acute lung injury: Giles F. Filley Lecture. Chest.

[12] Mestas J, Hughes CC (2004). Of mice and not men: differences between mouse and human immunology. J Immunol.

[13] Nourshargh S, Alon R (2014). Leukocyte migration into inflamed tissues. Immunity.

[14] Sengelov H, Kjeldsen L, Borregaard N (1993). Control of exocytosis in early neutrophil activation. J Immunol.

[15] Forner A, Reig M, Bruix J (2018). Hepatocellular carcinoma. Lancet.

[16] Anoopkumar-Dukie S, Carey JB, Conere T, O'Sullivan E, van Pelt FN, Allshire A (2005). Resazurin assay of radiation response in cultured cells. Br J Radiol.

[17] Ryschich E, Kerkadze V, Lizdenis P, Paskauskas S, Knaebel HP, Gross W, Gebhard MM, Buchler MW, Schmidt J (2006). Active leukocyte crawling in microvessels assessed by digital time-lapse intravital microscopy. J Surg Res.

[18] Paskauskas S, Parseliunas A, Kerkadze V, Nobiling R, Schmidt J, Ryschich E. Blockade of leukocyte haptokinesis and haptotaxis by ketoprofen, diclofenac and SC-560. BMC immunology. 2011;12(64). 10.1186/1471-2172-12-64 PMC3247092 22078067

[19] Knight MM, Roberts SR, Lee DA, Bader DL (2003). Live cell imaging using confocal microscopy induces intracellular calcium transients and cell death. Am J Phys Cell Phys.

[20] Beghetto C, Renken C, Eriksson O, Jori G, Bernardi P, Ricchelli F (2000). Implications of the generation of reactive oxygen species by photoactivated calcein for mitochondrial studies. Euro J Biochem.

[21] Nikfarjam M, Malcontenti-Wilson C, Fanartzis M, Daruwalla J, Christophi C (2004). A model of partial hepatectomy in mice. J Investig Surg.

[22] Takeichi T, Engelmann G, Mocevicius P, Schmidt J, Ryschich E (2010). 4-dimensional intravital microscopy: a new model for studies of leucocyte recruitment and migration in hepatocellular cancer in mice. J Gastrointest Surg.

[23] Winkler N, Strubing F, Gross W, Mier W, Ryschich E. Phenomenon of endothelial antibody capture: Principles and potential for locoregional targeting of hepatic tumors. Hepatology. 2018; 68(5): 1804-16. 10.1002/hep.30072 29734469

[24] Qian B, Kyuno D, Schafer M, Gross W, Mehrabi A, Ryschich E (2018). Liver segment imaging using monocyte sequestration: a potential tool for fluorescence-guided liver surgery. Theranostics.

[25] Qian B, Strubing F, Wang Z, Mehrabi A, Ryschich E (2018). Microsurgical technique of locoregional injection into the hepatic artery in tumor-bearing mice. Eur Surg Res.

[26] Yang L, Froio RM, Sciuto TE, Dvorak AM, Alon R, Luscinskas FW (2005). ICAM-1 regulates neutrophil adhesion and transcellular migration of TNF-alpha-activated vascular endothelium under flow. Blood.

[27] Yagublu V, Caliskan N, Lewis AL, Jesenofsky R, Gasimova L, Lohr JM, Keese M (2013). Treatment of experimental pancreatic cancer by doxorubicin-, mitoxantrone-, and irinotecan-drug eluting beads. Pancreatology.

[28] Bour G, Martel F, Goffin L, Bayle B, Gangloff J, Aprahamian M, Marescaux J, Egly JM (2014). Design and development of a robotized system coupled to mu CT imaging for intratumoral drug evaluation in a HCC mouse model. PLoS One.

[29] Ryschich E, Kerkadze V, Deduchovas O, Salnikova O, Parseliunas A, Marten A, Hartwig W, Sperandio M, Schmidt J (2009). Intracapillary leucocyte accumulation as a novel antihaemorrhagic mechanism in acute pancreatitis in mice. Gut.

[30] Castro-Santa E, Salnikova O, Ryschich E (2013). The role of beta2-integrins and CD44 in intrahepatic leucocyte sequestration. J Surg Res.

